# Neural Terms, International and National

**Published:** 1897-05

**Authors:** 


NEURAL TERMS, INTERNATIONAL AND NATIONAL. A Treatise on the Principles and Practice of Anatomic Nomenclature, with Special Reference to the Brain. By BURT G. WILDER, M. D., Professor of Neurology, etc., in Cornell University. Reprinted from the *Journal of Comparative Neurology*, VI., December, 1896. Pp. 137, including seven tables. Ithaca, N. Y.: Henry Cowell. 1897.

The many friends of nomenclature reform will hail with delight Dr. Wilder's treatise on neural terms. No one in America, perhaps not even in the world, has devoted so much time, energy and perseverance to this important but tedious work, and it is most gratifying to Dr. Wilder's many admirers to see his views so generally adopted not alone by American scientists, but European scientists as well. The treatise before us takes up historically the progress of the contest against ambiguity in neural and medical terms, and in most instances the success is attested by the adoption of the terms suggested by scientific bodies, as the American Society of Anatomists, the American Neurological Association, and others by the leading neurologists of Europe and America.

This monograph is an important addition to our knowledge of neuronymy and should be carefully read by all interested in the reform of our anatomical nomenclature. W. C. K.

